# Identification of pro-inflammatory CD205^+^ macrophages in livers of hepatitis B virus transgenic mice and patients with chronic hepatitis B

**DOI:** 10.1038/srep46765

**Published:** 2017-04-24

**Authors:** Liang Yong, Minmin Li, Yimin Gao, Yanru Deng, Wenbin Liu, Dake Huang, Cuiping Ren, Miao Liu, Jijia Shen, Xin Hou

**Affiliations:** 1 Anhui Provincial Laboratory of Microbiology and Parasitology; Department of Microbiology and Parasitology, Anhui Medical University, Hefei, China; 2 Intensive Care Unit, Affiliated Provincial Hospital of Anhui Medical University, Hefei, China; 3 Department of Hepatic Surgery and Anhui Province Key Laboratory of Hepatopancreatobiliary Surgery, Affiliated Provincial Hospital of Anhui Medical University, Hefei, China; 4 Integrated laboratory of College of Basic Medicine, Anhui Medical University, Hefei, China; 5 Department of Immunology, Anhui Medical University, Hefei, China

## Abstract

Hepatic macrophages play a central role in disease pathogenesis during hepatitis B virus (HBV) infection. Our previous study found that CD205^+^ macrophages in the liver of hepatitis B surface antigen transgenic (HBs-Tg) mice increased significantly compared with those in wild-type mice, and these increased CD205^+^ macrophages were involved in CpG-oligodeoxynucleotide-induced liver injury in HBs-Tg mice. Here, we analysed the phenotype and function of CD205^+^ macrophages derived from the liver of HBs-Tg mice and patients with chronic hepatitis B (CHB). We found that HBs-Tg mice-derived hepatic macrophages produced larger amounts of pro-inflammatory cytokines, including IL-6, IL-12, TNF-α, and of the anti-inflammatory cytokine IL-10 after stimulation with CpG-oligodeoxynucleotides or commensal bacteria DNA than B6 mice-derived hepatic macrophages. Furthermore, hepatic CD205^+^ macrophages from HBs-Tg mice showed an activated phenotype and expressed higher levels of inflammatory cytokine genes, chemokine genes, and phagocytosis-related genes than hepatic CD205^−^ macrophages. In addition, CD205^+^ macrophages displayed an inflammatory phenotype and were increased in the liver of patients with CHB compared with those in healthy controls. Our data suggest that hepatic CD205^+^ macrophages are a unique pro-inflammatory subset observed during HBV infection. Thus, development of intervention targeting these cells is warranted for immunotherapy of HBV-induced liver diseases.

Hepatitis B virus (HBV) infection may result in acute and chronic hepatitis, cirrhosis, and hepatocellular carcinoma. These disease progressions are driven by sustained inflammation. Immune cells, including CD8^+^ T cells, Th17 cells, regulatory T cells, dendritic cells, macrophages, natural killer (NK) cells, and NKT cells, modulate the inflammatory process during HBV infection^1^,^2^. Hence, better understanding of these immune cells will be important for developing effective treatment strategies for HBV-caused liver diseases.

Kupffer cells are the largest population of immune cells in the liver. They are the first barrier against pathogens entering the liver via the portal vein and are equipped with specific pattern recognition receptors, including toll-like receptors, C-type lectins, Nod-like receptors, RIG-like receptors, and scavenger receptors^3^. Kupffer cells play critical roles in the clearance of pathogens or cellular debris, maintenance of immunological tolerance in a steady state condition, and initiation as well as perpetuation of chronic inflammation and associated liver pathology^4^,^5^.

The functions of Kupffer cells during HBV infections are seemingly contradictory. Several studies have shown that upon HBV exposure Kupffer cells are activated to produce pro-inflammatory cytokines and chemokines and subsequently inhibit HBV replication in primary hepatocytes^6^,^7^. However, other studies have found that the signalling of pathogen recognition receptors and secretion of pro-inflammatory cytokines by Kupffer cells are inhibited by HBV, and the secretion of immunoregulatory cytokines and expression of membrane-bound inhibitory ligands by Kupffer cells are induced by HBV particles^8^,^9^,^10^,^11^. These studies suggest that Kupffer cells are essential for inhibiting the development of effective anti-viral immunity. The opposing effects of Kupffer cells in HBV infection may due to the infiltration of a large number of monocytes into the infected liver, resulting in heterogeneous subsets of macrophages. The various macrophage subsets fulfil diverse functions^12^. Recent studies in mice have demonstrated that Kupffer cells are critical for sensing liver injury and initiating inflammation, whereas infiltrating Ly6C^hi^ monocyte-derived macrophages elicit chronic liver injury and fibrosis. These Ly6C^hi^ macrophages can then differentiate into Ly6C^lo^ restorative macrophages and promote injury and fibrosis resolution^13^. Distinct macrophage populations have also been found in human liver, including the classical CD14^++^CD16^−^, intermediate CD14^++^CD16^+^, and nonclassical CD14^+^CD16^++^ monocytes/macrophages. The CD14^++^CD16^+^ monocytes/macrophages release abundant pro-inflammatory cytokines and directly activate collagen-producing hepatic stellate cells in patients during liver disease progression^14^,^15^,^16^.

CD205 is an endocytic type I C-type lectin-like molecule belonging to the mannose receptor family. It is expressed in a variety of cells, including dendritic cells, B cells, T cells and macrophages^17^,^18^. Most recently, CD205 has been identified as a cell surface receptor for CpG-oligodeoxynucleotides (ODNs)^19^. Our recent study showed that a population of CD205^+^ macrophages accumulated in the liver of hepatitis B surface antigen transgenic (HBs-Tg) mice. These CD205^+^ macrophages contributed to HBs-Tg mice having greater amounts of IL-12 and severer liver injury than B6 mice^20^. In the present study, we characterised CD205^+^ macrophages in the liver of HBs-Tg mice and found that CD205^+^ macrophages had a more pro-inflammatory phenotype and function than CD205^−^ macrophages. Furthermore, we confirmed that CD205^+^ monocytes/macrophages in the blood and liver of HBV-infected patients displayed an inflammatory phenotype and were increased relative to those in healthy controls.

## Results

### Hepatic macrophages in HBs-Tg mice are more sensitive to bacterial DNA stimulation than those in B6 mice

**Figure 1:**
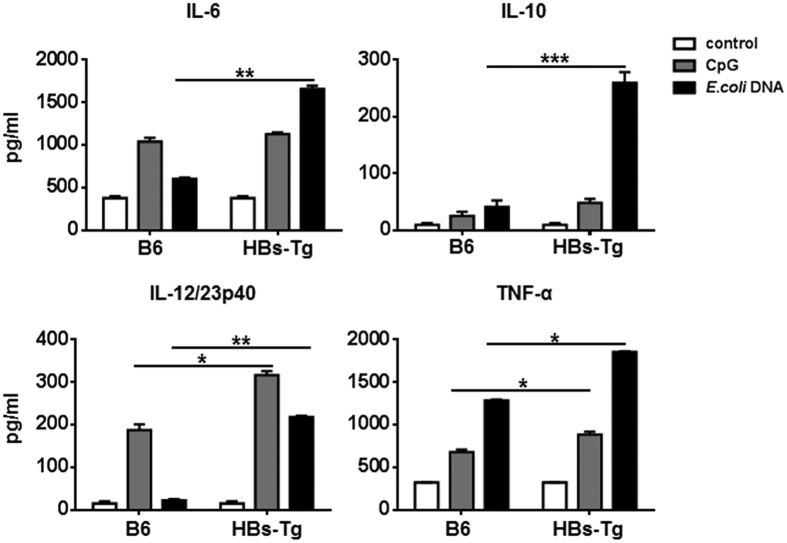
Inflammatory cytokines produced by hepatic macrophages in HBs-Tg and B6 mice. Hepatic macrophages were isolated from HBs-Tg (n = 5) and B6 (n = 5) mice and enriched using EasySep^TM^ Mouse Monocyte Enrichment Kit. Cells were stimulated with CpG (1 μM) or *E. coli* DNA (50 μg/mL) for 24 hours. Cytokines were measured by ELISA. Data represent mean ± SD from two independent experiments. *p < 0.05, **p < 0.01, ***p < 0.001, two-tailed unpaired Student’s *t*-test.

We previously reported that the levels of macrophage-derived cytokines IL-12 and TNF-α were higher in the serum of HBs-Tg mice than those in B6 mice after treatment with CpG-ODNs^20^, which suggests that hepatic macrophages in HBs-Tg mice are more sensitive to bacterial DNA. To confirm this in the present study, we isolated hepatic macrophages from HBs-Tg and B6 mice and assessed the function of the cytokines produced after stimulation with CpG-ODNs or commensal *Escherichia coli (E. coli*) DNA. Hepatic macrophages derived from HBs-Tg mice produced larger amounts of pro-inflammatory cytokines, including IL-6, IL-12/23p40, and TNF-α, as well as a greater amount of the anti-inflammatory cytokine IL-10 than those from B6 mice (Fig. 1).

### Hepatic CD205^+^ macrophages derived from HBs-Tg mice display an activated phenotype

**Figure 2:**
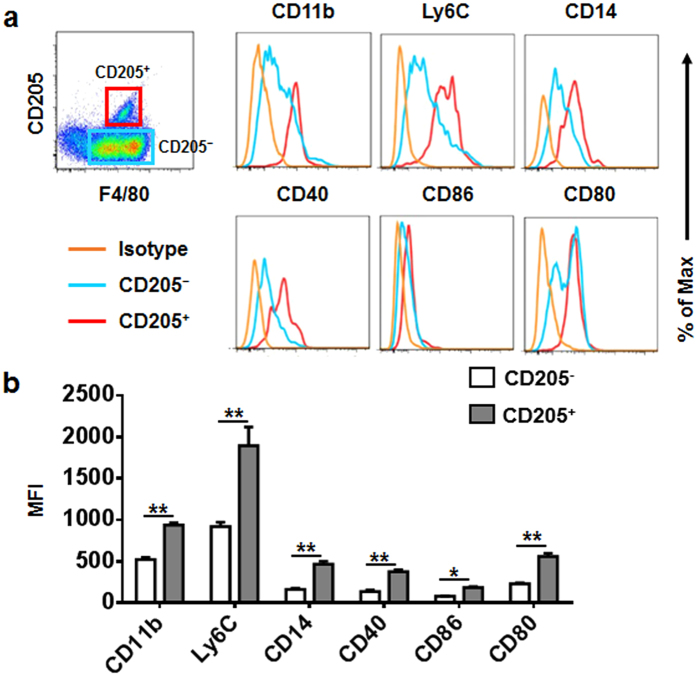
Expression of activation makers on hepatic macrophages in HBs-Tg mice. (**a**) Expression of CD11b, Ly6C, CD14, CD40, CD80, and CD86 on hepatic CD205^+/−^ macrophages in HBs-Tg mice (n = 4). (**b**) Statistical analysis of mean fluorescence intensity (MFI) of the markers shown in (**a**). Data are expressed as mean ± SD, and *p* values were calculated using two-tailed unpaired Student’s *t*-test. *p < 0.05, **p < 0.01 for the indicated comparisons.

CD205 can directly bind unmethylated CpG and enhance its uptake. We previously reported that compared with B6 mice, HBs-Tg mice had a greater percentage of hepatic CD205^+^ macrophages^20^. Thus, we speculated that CD205^+^ macrophages are a pro-inflammatory subset and contribute to the larger amounts of pro-inflammatory cytokine production observed in HBs-Tg mice. To test this hypothesis in the present study, we used flow cytometric analysis to assess the expression of several activation- or inflammation-associated molecules on hepatic CD205^+/−^ macrophages derived from HBs-Tg mice. Expression levels of the costimulatory molecules CD40, CD80, and CD86 on CD205^+^ macrophages were higher than those on CD205^−^ macrophages (Fig. 2a,b). Expression levels of CD14, an important coreceptor of toll-like receptors^21^, were also increased on CD205^+^ macrophages (Fig. 2a,b). In addition, CD205^+^ macrophages expressed high levels of CD11b and Ly6C (Fig. 2a,b), indicating that CD205^+^ macrophages may be derived from inflammatory monocytes. These data suggest that hepatic CD205^+^ macrophages in HBs-Tg mice are more activated than CD205^−^ macrophages.

### Hepatic CD205^+^ macrophages in HBs-Tg mice show an inflammatory gene expression profile

**Figure 3:**
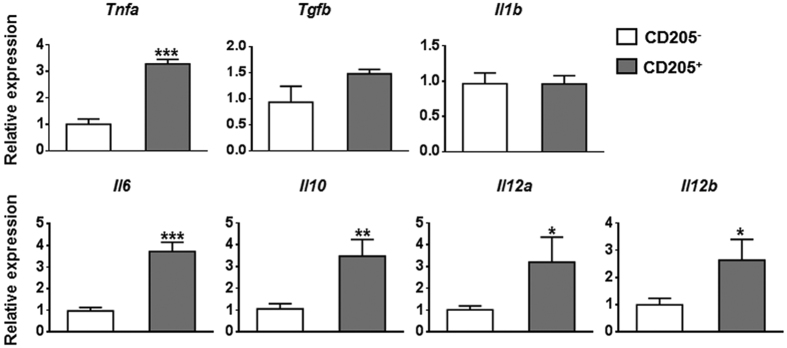
Cytokine gene expression profiles of hepatic CD205^+^ and CD205^−^ macrophages. Hepatic macrophage subsets were isolated from HBs-Tg mice by FACS sorting. Quantitative PCR analysis compared mRNA expression levels of cytokine genes between hepatic CD205^+^ and CD205^−^ macrophages. Data are normalised to *Actb* in the same sample and expressed as fold change in comparison with CD205^−^ macrophages. Data represent mean ± SD from three independent experiments. *p < 0.05, **p < 0.01, ***p < 0.001, two-tailed unpaired Student’s *t*-test.

**Figure 4:**
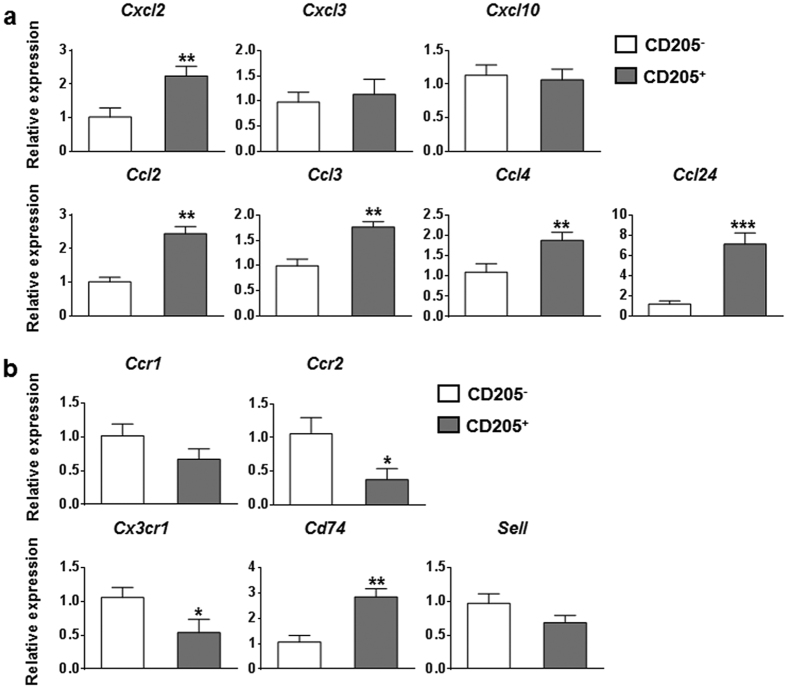
Expression of chemokine and chemokine receptor genes in hepatic CD205^+^ and CD205^−^ macrophages. Quantitative PCR analysis of relative mRNA levels of chemokine genes (**a**) and chemokine receptor genes (**b**) in hepatic CD205^+^ and CD205^−^ macrophages derived from HBs-Tg mice. Values are expressed as fold amplification over CD205^−^ macrophages following normalization with *Actb*. Data represent mean ± SD from three independent experiments. *p < 0.05, **p < 0.01, ***p < 0.001, two-tailed unpaired Student’s *t*-test.

**Figure 5:**
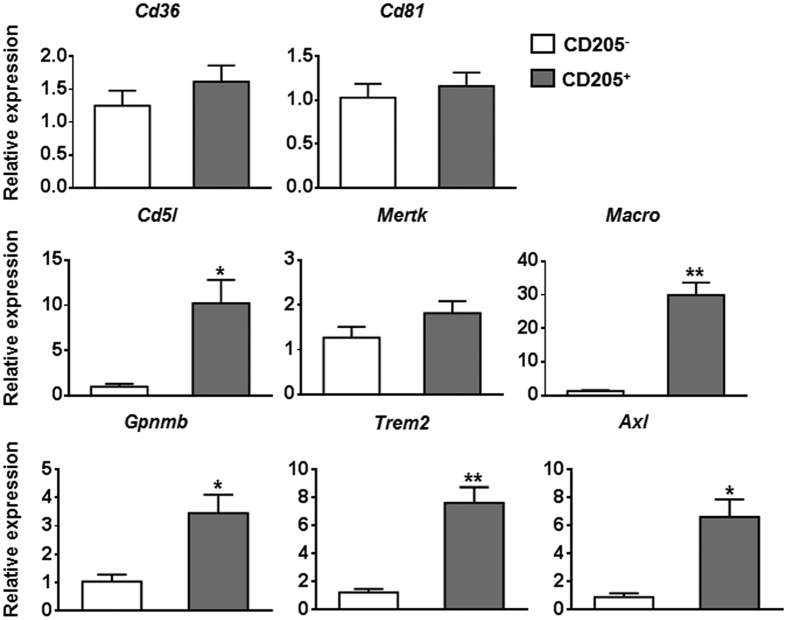
Hepatic CD205^+^ macrophages in HBs-Tg mice overexpress phagocytosis-related genes. Quantitative PCR analysis comparing mRNA levels of phagocytosis-related genes between hepatic CD205^+^ and CD205^−^ macrophages in HBs-Tg mice. Values are expressed as fold amplification over CD205^−^ macrophages following normalization with *Actb*. Data represent mean ± SD from three independent experiments. *p < 0.05, **p < 0.01, two-tailed unpaired Student’s *t*-test.

To further determine the inflammatory characteristics of hepatic CD205^+^ macrophages in HBs-Tg mice, we sorted hepatic CD205^+^ macrophages and CD205^−^ macrophages derived from the liver of HBs-Tg mice. The purity of purified cells was confirmed to be more than 96% (Supplementary Fig. S1). We then used quantitative PCR to analyse the expression levels of cytokine genes, chemokine genes, chemokine receptor genes and phagocytosis-related genes. Compared with those in CD205^−^ macrophages, inflammatory cytokine genes, including *Tnfa, Il6*, and *Il12*, were upregulated in CD205^+^ macrophages. The gene expression of *Il10* was also increased in CD205^+^ macrophages (Fig. 3). The levels of most of the chemokine genes we tested, including *Cxcl2, Ccl2, Ccl3, Ccl4*, and *Ccl24*, were upregulated in CD205^+^ macrophages compared with those in CD205^−^ macrophages (Fig. 4a). The expression levels of chemokine receptor genes varied, for example, *Ccr2* and *Cx3cr1* were downregulated, whereas *Cd74* was upregulated in CD205^+^ macrophages compared with those in CD205^−^ macrophages (Fig. 4b). A number of phagocytosis-related genes, such as *Cd5l, Macro, Gpnmb, Trem2*, and *Axl*, were upregulated in CD205^+^ macrophages compared with those in CD205^−^ macrophages (Fig. 5). These quantitative PCR results suggest that CD205^+^ macrophages are pro-inflammatory and have a stronger ability than CD205^−^ macrophages to phagocytose.

### Peripheral CD205^+^ monocytes are increased in hepatitis B surface antigen (HBsAg)-positive donors

**Figure 6:**
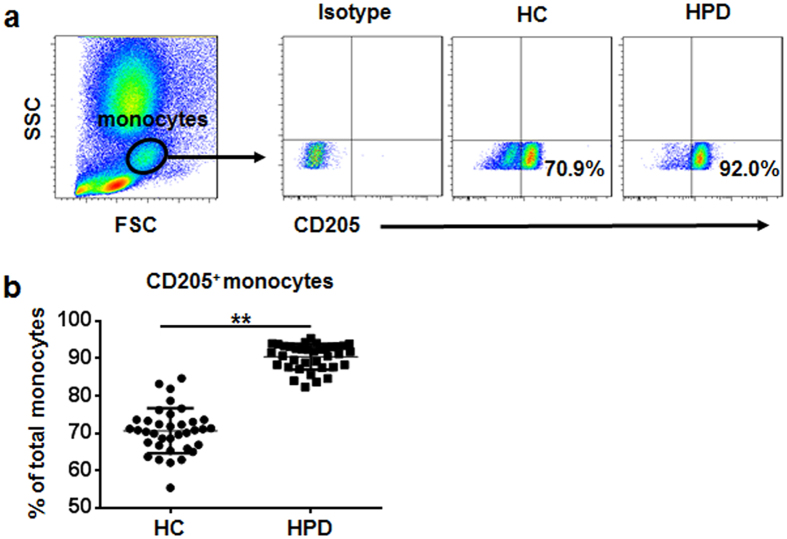
Percentage of peripheral CD205^+^ monocytes is markedly increased in HBsAg-positive donors. (**a**) Monocyte subsets in peripheral blood were gated based on forward and side scatter (left panel), and CD205 expression on peripheral monocytes of HBsAg-positive donors (HPD) and healthy controls (HC) (right panel). (**b**) Pooled data show the percentages of CD205^+^ monocyte subset in HPD (n = 36) and HC (n = 36). Data are expressed as mean ± SD. **p < 0.01, two-tailed unpaired Student’s *t*-test.

We next explored CD205 expression on peripheral monocytes in HBsAg-positive donors (HPD) and healthy controls. As shown in Fig. 6, compared with healthy controls, HPD showed an increased percentage of peripheral CD205^+^ monocytes.

### Identification of CD205^+^ macrophages in the liver of patients with chronic hepatitis B

**Figure 7:**
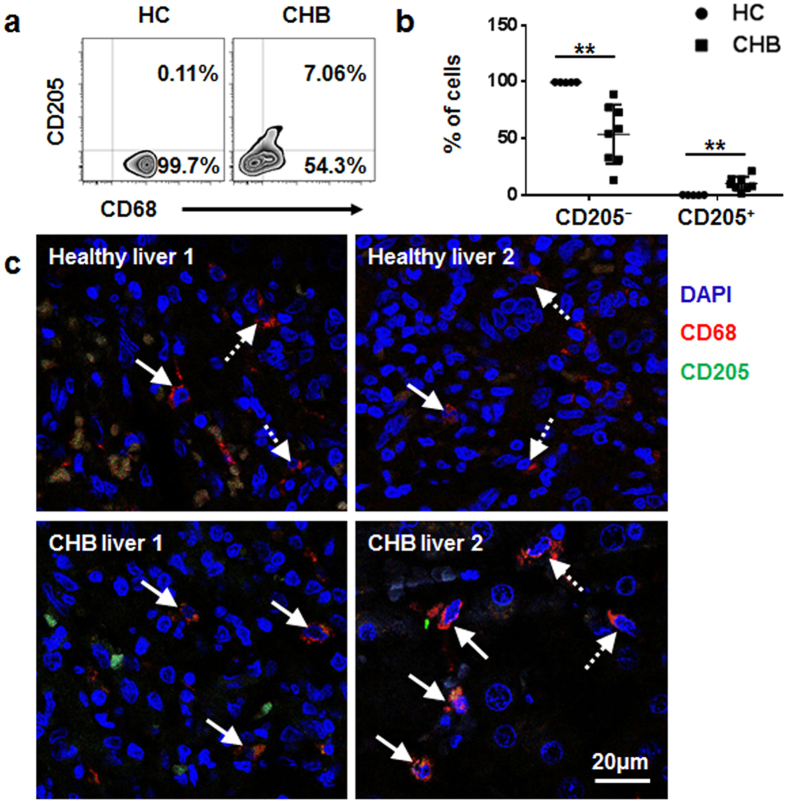
CD205^+^ macrophages are increased in the liver of patients with CHB. (**a**) A representative graph of hepatic CD68^+^CD205^+/−^ macrophages in patients with CHB and healthy controls (HC). (**b**) Individual values of proportions of hepatic CD68^+^CD205^+/−^ macrophages in patients with CHB (n = 8) and HC (n = 5). Data are expressed as mean ± SD, and *p* values were calculated using two-tailed unpaired Student’s *t*-tests. **p < 0.01. (**c**) Immunofluorescence microscopy images of human livers from two patients with CHB and two HC stained with anti-CD205 (green), anti-CD68 (red), and DAPI (blue). The solid arrows show CD68^+^CD205^+^ macrophages, and the dashed arrows show CD68^+^CD205^−^ macrophages.

**Figure 8:**
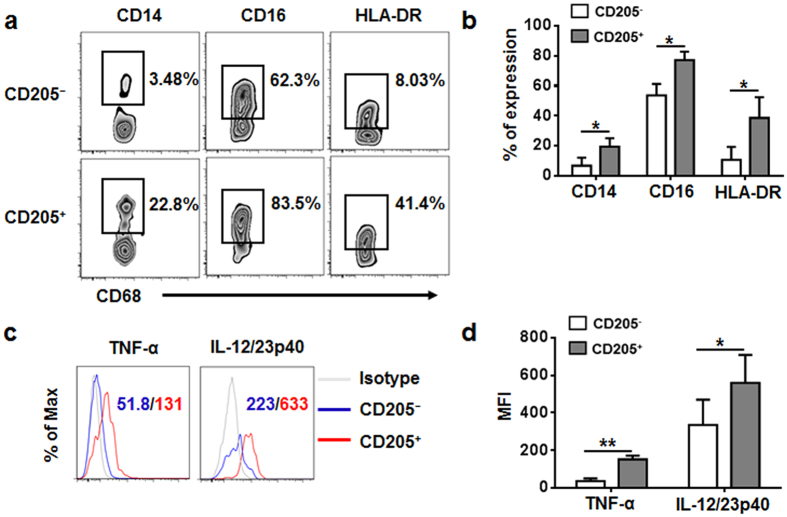
Identification of CD205^+^ macrophages in the liver of patients with CHB. (**a**,**b**) Expression of CD14, CD16, and HLA-DR on hepatic CD205^+/−^ macrophages derived from patients with CHB (n = 4). (**c**,**d**) Hepatic leukocytes were stimulated with PMA (30 ng/mL) and ionomycin (1 μg/mL) for 4 hours in the presence of monensin. Intracellular expression levels of TNF-α and IL-12/23p40 in hepatic CD205^+^ and CD205^−^ macrophages derived from patients with CHB (n = 5) were detected by flow cytometry. Data are expressed as mean ± SD. *p < 0.05, **p < 0.01, two-tailed unpaired Student’s *t*-test.

We also analysed CD205 expression on hepatic macrophages derived from patients with chronic hepatitis B (CHB) and from healthy controls by using flow cytometry and immunofluorescence analyses. We observed a significant increase of CD205^+^ macrophages in the liver of patients with CHB compared with those in healthy controls (Fig. 7a–c). We then identified the phenotypic features of hepatic CD205^+^ macrophages in patients with CHB. We found that the levels of CD14, CD16, and HLA-DR on CD205^+^ macrophages were higher than those on CD205^−^ macrophages (Fig. 8a,b). In addition, CD205^+^ macrophages displayed increased secretion of TNF-α and IL-12/23p40 compared with CD205^−^ macrophages (Fig. 8c,d).

## Discussion

During HBV infection, Kupffer cells, the liver-resident macrophages, are activated and proliferate^1^, and a large number of monocytes infiltrate into the infected liver. Their differing origins make hepatic macrophages a heterogeneous population of immune cells that perform diverse functions^12^,^22^. In the present study, we identified and characterised a unique pro-inflammatory CD205^+^ macrophage subset in the HBV-infected liver. This CD205^+^ macrophage population accumulated in the livers of HBs-Tg mice and patients with CHB, and was highly activated, secreting high levels of cytokines and chemokines, suggesting that it contributes to the sustained inflammation in HBV-infected livers.

It has been reported that resident Kupffer cells in murine liver are F4/80^hi^CD11b^intermediate^, whereas recruited monocyte-derived macrophages are CD11b^hi^F4/80^intermediate^ 13,23. CD205^+^ macrophages in the liver of HBs-Tg mice expressed high levels of CD11b and intermediate levels of F4/80 (Fig. 2); moreover, both the total hepatic macrophage number and CD205^+^ macrophage number in HBs-Tg mice were higher than those in B6 mice (Supplementary Fig. S2); thus, we speculated that these cells arise from circulating monocytes. Ly6C has been widely used to identify functionally distinct populations of circulating murine monocytes and macrophages in diseased tissues: Ly6C^hi^ monocytes/macrophages are thought to initiate inflammatory activities, whereas Ly6C^lo^ monocytes/macrophages promote healing^13^,^24^,^25^. The CD205^+^ macrophages examined in the present study expressed high levels of Ly6C, indicating that they are a pro-inflammatory subset. Our flow cytometric analysis of costimulatory molecules (including CD40, CD80, and CD86) on hepatic CD205^+/−^ macrophages confirmed that CD205^+^ macrophages were more activated than CD205^−^ macrophages. Hepatic CD205^+^ macrophages derived from both HBs-Tg and B6 mice display a more activated phenotype than that of CD205^−^ macrophages (Supplementary Fig. S2). In the healthy liver of B6 mice, theses CD205^+^ macrophages may be involved in persistent and regulated inflammation caused by constantly changing metabolism, tissue remodeling activity and commensal bacterial products. Because of the specific liver microenvironment of HBs-Tg mice, hepatic CD205^+^ macrophages in HBs-Tg mice were much more than those in B6 mice.

Through quantitative PCR analysis, we found that CD205^+^ macrophages had higher expression levels than CD205^−^ macrophages of inflammatory cytokine genes, including *Tnfa, Il6*, and *Il12* (Fig. 3). TNF-α and IL-6 are important mediators of liver damage^26^,^27^. They also induce fibrogenesis by activating hepatic stellate cells^28^,^29^. IL-12 can promote the activation of NK, NKT, and T cells, which are essential executors of hepatic injury^20^,^30^,^31^. These data suggest that CD205^+^ macrophages are associated with liver injury and fibrosis during HBV infection. However, compared with CD205^−^ macrophages, CD205^+^ macrophages displayed higher mRNA expression levels of IL-10, which has a general suppressive effect and plays an important role in the prevention of tissue damage and fibrogenesis^32^. The upregulation of IL-10 may be part of a self-regulating negative feedback mechanism to avoid overactivation.

Many chemokine genes, including *Cxcl2, Ccl2, Ccl3, Ccl4*, and *Ccl24*, were upregulated in CD205^+^ macrophages compared with CD205^−^ macrophages (Fig. 4a). Macrophage-secreted chemokines can recruit innate and adaptive immune cells (such as NK cells, NKT cells, dendritic cells, and T cells) in the liver. In turn, NK/NKT cells and T cells produce cytokines, such as TNF-α and IFN-γ, leading to further macrophages activation and hepatocyte damage^6^,^33^,^34^. Thus, CD205^+^ macrophages contribute to inflammation in the liver by not only secreting cytokines but also producing chemokines. In addition, CD205^+^ macrophages likely had high levels of phagocytic activity because phagocytosis-related genes, which are associated with the recognition, binding, and clearance of pathogens and apoptotic cells, were upregulated in CD205^+^ macrophages (Fig. 5). Taken together, our results indicate that CD205^+^ macrophages in HBs-Tg mice share some phenotypic and functional features with the profibrotic Ly6C^hi^ macrophages derived from the liver of carbon tetrachloride (CCl_4_)-treated mice, with respect to high mRNA levels of inflammatory cytokines and chemokines^13^. However, the CD205^+^ macrophages examined here also expressed high levels of phagocytosis-related genes, which are low in Ly6C^hi^ macrophages in the CCl_4_ model of liver fibrosis. The differences between these two populations of macrophages may be due to the different liver disease microenvironments. Thus, compared with profibrotic Ly6C^hi^ macrophages, CD205^+^ macrophages are more similar to human intrahepatic CD14^+^CD16^+^ monocytes/macrophages, which possess high phagocytic activity and secrete pro-inflammatory and profibrogenic cytokines and chemokines^14^.

We confirmed our findings in the liver of patients with CHB. We determined that the percentage of hepatic CD205^+^ macrophages in patients with CHB was greater than that in healthy controls. CD205^+^ macrophages exhibited higher HLA-DR expression and produced higher levels of TNF-α and IL-12 than CD205^−^ macrophages, indicating that CD205^+^ macrophages are highly activated and may be closely related to the liver damage in patients with CHB. In addition, our results demonstrated that CD205^+^ macrophages partly overlapped with CD14^+^CD16^+^ monocytes/macrophages in the liver: approximately 20% of CD205^+^ macrophages were CD14^+^CD16^+^, and approximately 50% of CD14^+^CD16^+^ monocytes/macrophages were CD205^+^. Therefore, CD205^+^ macrophages and CD14^+^CD16^+^ monocytes/macrophages are different subsets with similar phenotypic and functional features in the liver.

In conclusion, we identified features of a CD205^+^ macrophage subset in the livers of HBs-Tg mice and patients with CHB, and suggested that CD205^+^ macrophages are associated with inflammatory responses and liver damage in these patients. Thus, CD205^+^ macrophages are a potential therapeutic target in HBV-caused liver diseases.

## Methods

### Subjects

Peripheral blood of healthy controls and HBsAg-positive donors came from First Affiliated Hospital of Anhui Medical University (Hefei, China). Human liver samples derived from patients with CHB and healthy controls listed in Table 1 were obtained from Affiliated Provincial Hospital of Anhui Medical University (Hefei, China). Patients with CHB were characterized by the presence of HBsAg and HBV DNA. Uninfected healthy liver samples came from surgical resections of uninvolved liver tissues of angeioma patients. The informed consent was obtained from all subjects. All experiments were approved by Institutional Ethical Committee of Anhui Medical University, and were performed in accordance with the principles of the Declaration of Helsinki including any relevant details.

### Experimental animals

The 8–10 week-old male HBs-Tg mice C57BL/6J-TgN (AlblHBV) 44Bri (H-2b, containing partial HBV genome, including S, pre-S and X genes) were purchased from the Vital River experimental animal company (Beijing, China). HBs-Tg mice were originally from the Jackson Laboratory (Bar Harbor, ME, USA). Age- and sex-matched wild-type littermates were used. All mice were fed in a specific pathogen-free environment. All animal experiments were approved by the Institutional Animal Care and Use Committee at Anhui Medical University and conformed to the guidelines outlined in the Guide for the Care and Use of Laboratory Animals.

### Cell isolation

A two-step collagenase perfusion method as described previously was used to isolate murine hepatic macrophages^20^. The collected cells were used for flow cytometric analysis. For culture of murine hepatic macrophages, collected cells were further enriched using EasySep^TM^ Mouse Monocyte Enrichment Kit (Stemcell Technologies). HBs-Tg mice-derived hepatic (DAPI^−^CD45^+^CD11b^+^F4/80^+^) CD205^+^ and CD205^−^ macrophage subsets were isolated by FACS (BECKMAN COULTER MoFlo Astrios EQ). And the purity was >96%.

To enrich human peripheral leukocytes, blood samples were incubated with ACK Lysis Buffer (0.829% NH_4_Cl, 0.1% KHCO_3_, 0.00372% EDTA-2Na, pH 7.2–7.4) to remove red blood cells.

For isolation of human hepatic leukocytes, liver samples were cut with scissors and incubated in DMEM containing 0.05% Collagenase IV (Sigma) and 0.002% DNase I (Sangon Biotch) at 37 °C for 60 minutes. Then, the viable cells were separated by 40% Percoll (GE Healthcare) solution.

### Flow cytometric analysis

Cells were blocked with FcR-blocker (BD PharMingen), and then incubated with monoclonal Abs for surface antigens. Fluorochrome-conjugated monoclonal Abs used in this study were: anti-mouse F4/80, CD205, anti-human CD68, CD205, CD14, CD16, HLA-DR, TNF-α, IL-12/23p40 (BioLegend), anti-mouse CD45, CD11b, Ly6C, CD14, CD40, CD80, CD86 (BD PharMingen). Intracellular staining was performed using the Transcription Factor Staining Buffer Set Kit (eBioscience) and Abs to human CD68, TNF-α, IL-12/23p40. All samples were analysed using FACSVerse flow cytometry and FlowJo 7.6.1 software.

### RNA isolation and quantitative PCR

FACS-sorted hepatic CD205^+^ and CD205^−^ macrophages were resuspended in 1.0 mL Trizol (Invitrogen) and RNA was isolated according to the manufacturer’s instructions. First strand cDNA was synthesized from ≤500 ng of RNA using PrimeScript^TM^ RT reagent Kit (Perfect Real Time) (TaKaRa). Quantitative PCR was performed by Applied Biosystems StepOnePlus Real-Time PCR System with SYBR *Premix Ex Taq*^TM^ II (Tli RNaseH Plus) (TaKaRa). For analysis, all expression levels of target genes were normalized to the housekeeping gene *Actb*. Gene expression values were then calculated based on the 2^−ΔΔCt^ method. The primers were shown in Table 2.

### CpG-ODNs and *E. coli* DNA

Mouse CpG-ODNs (HC4033: 5′-TCCATGACGTTCCTGATGCT-3′) was purchased from HyCult Biotechnology (Uden, Netherlands) and was dissolved in pyrogen-free saline. *E. coli* DNA was isolated from *E. coli* (ATCC25922) using Bacterial Genomic DNA Isolation Kit (Solarbio) and incubated with Polymyxin B sulfate (50 μg/mL) at room temperature for 30 minutes for removing LPS.

### *In vitro* stimulation

Enriched hepatic macrophages derived from HBs-Tg and B6 mice were seeded on a 48-well cell culture plate (1 × 10^6^ cells/mL) in DMEM medium supplemented with 10% FBS and antibiotics (100 units/mL penicillin and 100 μg/mL streptomycin). Cells were stimulated with CpG-ODNs (1 μM) or *E. coli* DNA (50 μg/mL) for 24 hours. For measurement of cytokines, culture supernatants were kept and stored at −80 °C until the cytokine assay.

### Cytokine assay

Mouse IL-6, IL-10, IL-12/23p40 and TNF-α were measured using cytokine-specific enzyme-linked immunosorbent assay (ELISA) kits according to the manufacturer’s instructions (R&D system).

### Immunofluorescence

Formalin-fixed and paraffin-embedded human liver tissue sections were used for immunofluorescence. Deparaffinized sections were heated at 100 °C for 15 minutes in 0.01 M sodium citrate buffer (pH 6.0) in a microwave oven. Immunofluorescence blocking solution (Beyotime) was used to block nonspecific binding at 37 °C for 30 minutes. Sections were incubated with mouse anti-human CD68 antibody (Abcam) and rabbit anti-human LY75 (CD205) antibody (Abcam) at 4 °C overnight. Sections were washed by PBS and incubated with Rhodamine-conjugated goat anti-mouse IgG (H+L) (ZSGB-BIO) and FITC-conjugated goat anti-rabbit IgG (H+L) (ZSGB-BIO) at 37 °C for 30 minutes. After washing with PBS, sections were incubated with DAPI (Beyotime) to stain nuclei, and observed and photographed using confocal scanning microscope (LEICA SP5-DMI6000-DIC).

### Statistical analysis

All data were expressed as mean ± SD and analysed using GraphPad Prism 6.01 software. Two-tailed unpaired Student’s *t*-test was used to compare variables between two groups and one-way ANOVA with Bonferroni’s post-test was used for comparisons between more than two groups. *P* < 0.05 was considered to be significant.

## Additional Information

**How to cite this article:** Yong, L. *et al*. Identification of pro-inflammatory CD205^+^ macrophages in livers of hepatitis B virus transgenic mice and patients with chronic hepatitis B. *Sci. Rep.* **7**, 46765; doi: 10.1038/srep46765 (2017).

**Publisher's note:** Springer Nature remains neutral with regard to jurisdictional claims in published maps and institutional affiliations.

## Supplementary Material

Supplementary Information

## Figures and Tables

**Table 1:** Characteristics of healthy controls and CHB patients.

Group	Healthy controls	CHB patients
Total	9	11
Sex (male/female)	4/5	7/4
Median age (years)	45	55
ALT (U/L)	17 (6–30)	56 (16–79)
HBV DNA copies/mL	Negative	3.73 × 10^4^ (1.23–6.74 × 10^4^)

**Table 2:** Sequences of primers.

Genes	Forward primer (5′–3′)	Reverse primer (5′–3′)
*Actb*	AGAGGGAAATCGTGCGTGAC	CAATAGTGATGACCTGGCCGT
*Tnfa*	ACTGGCAGAAGAGGCACTC	CTGGCACCACTAGTTGGTTG
*Tgfb*	ACAATTCCTGGCGTTACCTT	AGCCCTGTATTCCGTCTCC
*Il1b*	CTGAACTCAACTGTGAAATGC	TGATGTGCTGCTGCGAGA
*Il6*	ACACATGTTCTCTGGGAAATCGT	AAGTGCATCATCGTTGTTCATACA
*Il10*	GCTCTTACTGACTGGCATGAG	CGCAGCTCTAGGAGCATGTG
*Il12a*	CTGTGCCTTGGTAGCATCTATG	GCAGAGTCTCGCCATTATGATTC
*Il12b*	TGGTTTGCCATCGTTTTGCTG	ACAGGTGAGGTTCACTGTTTCT
*Cxcl2*	ACCAACCACCAGGCTACA	TCAGGGTCAAGGCAAACT
*Cxcl3*	CCACCAACCACCAGGCTACA	GAGGCAAACTTCTTGACCATCC
*Cxcl10*	TCATCCCTGCGAGCCTATCC	TGCGTGGCTTCACTCCAGTT
*Ccl2*	CCAGCAAGATGATCCCAATG	TACGGGTCAACTTCACATTC
*Ccl3*	GATTCCACGCCAATTCATCG	AGGCATTCAGTTCCAGGTCA
*Ccl4*	TTTCTCTTACACCTCCCGGC	AGCTGCTCAGTTCAACTCCA
*Ccl24*	CTCCTTCTCCTGGTAGCCTG	ATGGCCCTTCTTGGTGATGA
*Ccr1*	CTCATGCAGCATAGGAGGCTT	ACATGGCATCACCAAAAATCCA
*Ccr2*	TGTGATTGACAAGCACTTAGACC	TGGAGAGATACCTTCGGAACTT
*Cx3cr1*	GAGTATGACGATTCTGCTGAGG	CAGACCGAACGTGAAGACGAG
*Cd74*	CCGCCTAGACAAGCTGACC	ACAGGTTTGGCAGATTTCGGA
*Sell*	TACATTGCCCAAAAGCCCTTAT	CATCGTTCCATTTCCCAGAGTC
*Cd36*	ATGGGCTGTGATCGGAACTG	GTCTTCCCAATAAGCATGTCTCC
*Cd81*	GTGGAGGGCTGCACCAAAT	GACGCAACCACAGAGCTACA
*Cd5l*	GATCGTGTTTTTCAGAGTCTCCA	TGCAGTCAACCCCTTGAATAAG
*Mertk*	CAGGGCCTTTACCAGGGAGA	TGTGTGCTGGATGTGATCTTC
*Macro*	ACAGAGCCGATTTTGACCAAG	CAGCAGTGCAGTACCTGCC
*Gpnmb*	CATTCCCATCTCGAAGGTGAAA	AAATGGCAGAGTCGTTGAGGA
*Trem2*	CTGGAACCGTCACCATCACTC	CGAAACTCGATGACTCCTCGG
*Axl*	ATGGCCGACATTGCCAGTG	CGGTAGTAATCCCCGTTGTAGA
