# Supplementary material for: Identification of pro-inflammatory CD205+ macrophages in livers of hepatitis B virus transgenic mice and patients with chronic hepatitis B
Source: Sci Rep. 2017 Apr 24;7:46765. doi: 10.1038/srep46765 (PMC5402278; doi:10.1038/srep46765)
Supplement: Supplementary Information [file srep46765-s1.pdf]

Identification of pro-inflammatory CD205$^+$ macrophages in livers of hepatitis B virus transgenic mice and patients with chronic hepatitis B

Liang Yong[1], Minmin Li[1], Yimin Gao[1], Yanru Deng[2], Wenbin Liu[3], Dake Huang[4], Cuiping Ren[1], Miao Liu[1], Jijia Shen[1], and Xin Hou[1,5]

[1]Anhui Provincial Laboratory of Microbiology and Parasitology; Department of Microbiology and Parasitology, Anhui Medical University, Hefei, China

[2]Intensive Care Unit, Affiliated Provincial Hospital of Anhui Medical University, Hefei, China

[3]Department of Hepatic Surgery and Anhui Province Key Laboratory of Hepatopancreatobiliary Surgery, Affiliated Provincial Hospital of Anhui Medical University, Hefei, China

[4]Integrated laboratory of College of Basic Medicine, Anhui Medical University, Hefei, China

[5]Department of Immunology, Anhui Medical University, Hefei, China

Correspondence to: Xin Hou, email: houxin45@mail.ustc.edu.cn

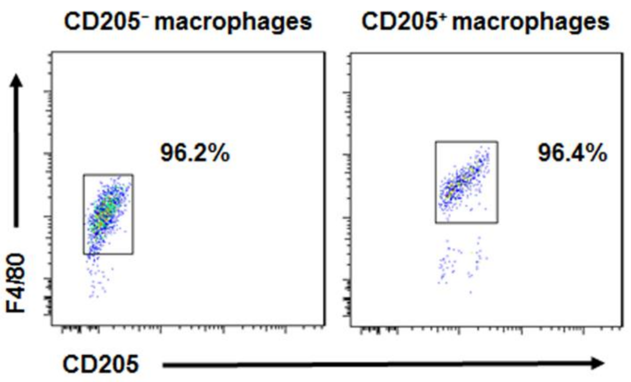

Supplementary Figure S1. The purity of hepatic CD205$^+$ and CD205$^-$ macrophages after sorting in HBs-Tg mice. Cells were gated on DAPI$^-$CD45$^+$CD11b$^+$F4/80$^+$ macrophages and analysed the expression of CD205.

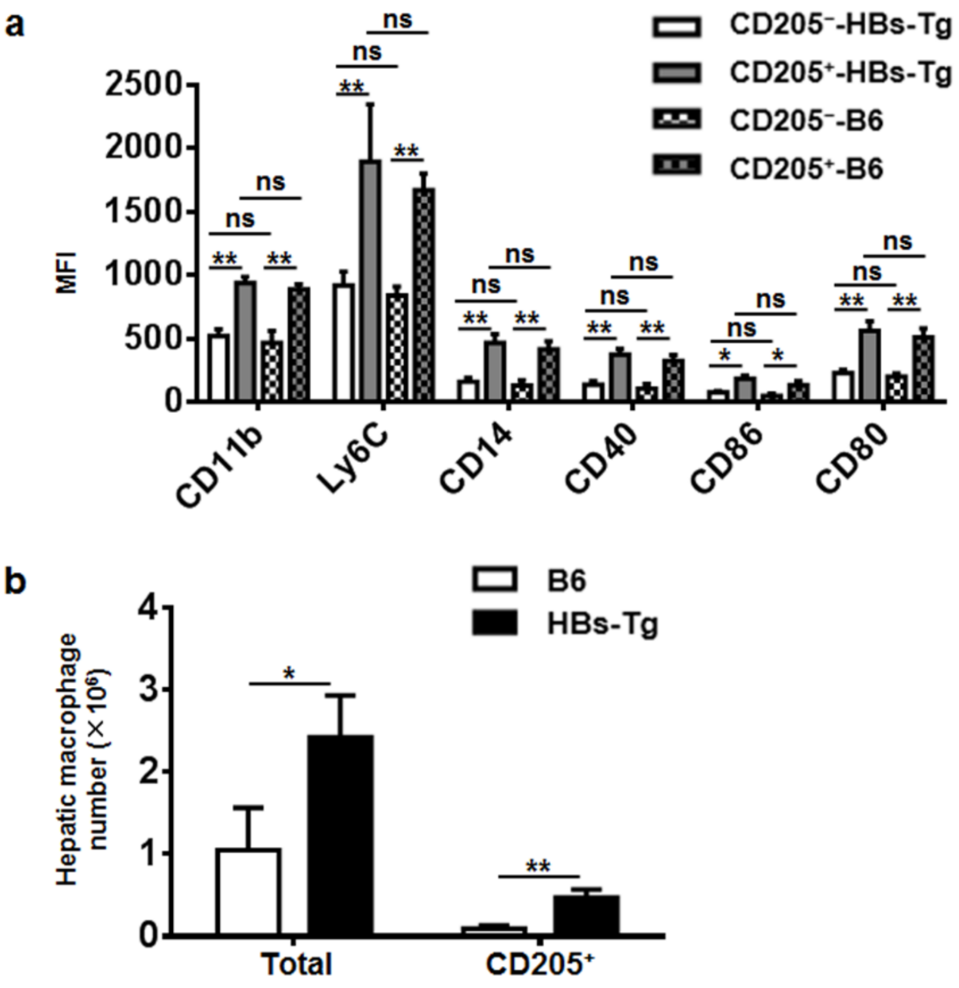

Supplementary Figure S2. Expression of activation makers on hepatic macrophages and hepatic macrophage number in HBs-Tg and B6 mice. (a)

Expression of CD11b, Ly6C, CD14, CD40, CD80, and CD86 on hepatic $CD205^{+/-}$ macrophages in HBs-Tg (n = 4) and B6 (n = 4) mice. Data are expressed as mean ± SD. *$p < 0.05$, **$p < 0.01$, ns, not significant, one-way ANOVA with Bonferroni's post-test. (**b**) Cell numbers of total hepatic macrophages and $CD205^{+}$ macrophages in HBs-Tg (n = 4) and B6 (n = 4) mice. Data are expressed as mean ± SD. *$p < 0.05$, **$p < 0.01$, two-tailed unpaired Student's *t*-test.
